# Molecular identification of natural hybridization between *Melastoma malabathricum* and *Melastoama beccarianum* in Sarawak, Malaysia

**DOI:** 10.1002/ece3.5160

**Published:** 2019-04-15

**Authors:** Renzhi Wu, Peishan Zou, Guangwen Tan, Zhenyang Hu, Yongqi Wang, Zulin Ning, Wei Wu, Ying Liu, Shaoyun He, Renchao Zhou

**Affiliations:** ^1^ College of Forestry and Landscape Architecture South China Agricultural University Guangzhou China; ^2^ State Key Laboratory of Biocontrol and Guangdong Key Laboratory of Plant Resources, School of Life Sciences Sun Yat‐sen University Guangzhou China; ^3^ Pubang Landscape Architecture Co., Ltd Guangzhou China; ^4^ Guangdong Provincial Key Laboratory of Applied Botany, South China Botanical Garden Chinese Academy of Sciences Guangzhou China

**Keywords:** introgression, *Melastoma*, molecular identification, natural hybridization, nuclear genes

## Abstract

Hybridization is very common in flowering plants and it plays a significant role in plant evolution and adaptation. *Melastoma* L. (Melastomataceae) comprises about 80–90 species in tropical Asia and Oceania, among which 41 species occur in Borneo. Natural hybridization is frequently reported in *Melastoma* in China, but so far there have been no confirmed cases of hybridization in Southeast Asia (including Borneo), where most species occur. Here, we identified a case of natural hybridization between *Melastoma malabathricum* L. and *Melastoma beccarianum* Cogn. in Sarawak, Malaysia, by using sequence data of three nuclear genes and one chloroplast intergenic spacer. *Melastoma malabathricum* is the most widespread species of this genus, occurring in almost the whole range of this genus, while *M. beccarianum* is a local species endemic to northern Borneo. Our results showed that natural hybridization and introgression occur between *M. malabathricum* and *M. beccarianum*, and the introgression was asymmetrical, mainly from *M. malabathricum* to *M. beccarianum*. As adaptive traits can be transferred by introgression, our study suggests that natural hybridization should be a significant mechanism for the evolution and adaptation of *Melastoma* in Southeast Asia. However, introgression from the common species *M. malabathricum* to the relatively rare species *M. beccarianum* may cause the decline of *M. beccarianum*, incurring conservation concern. With a large number of species of *Melastoma* and almost year‐around flowering in Southeast Asia, more cases of natural hybridization are expected to be found and identified in near future.

## 1 INTRODUCTION

Hybridization is referred to a process through which there is interbreeding between species of two genetically distinct populations or species (Harrison & Larson, 2014). Hybridization is very common in flowering plants and it plays a significant role in plant evolution and adaptation (Abbott et al., 2013; Arnold, 1997). The evolutionary outcomes of hybridization include hybrid speciation, reinforcement of prezygotic isolation, transfer of genetic materials between species (introgression), and so on (Abbott et al., 2013; Arnold, 1997). Introgression can have both positive and negative effects on genetic diversity and adaptation. On one hand, introgression from other species can contribute to the increase of genetic diversity and rapid adaptation to novel environments by transfer of adaptive alleles (Abbott, Barton, & Good, 2016; Lamichhaney et al., 2016; Zhang, Dasmahapatra, Mallet, Moreira, & Kronforst, 2016), on the other hand, introgressive hybridization between a common species and a rare species can cause genetic assimilation of the rare species by the common species and thus incurs the risk of extinction of the rare species (Huxel, 1999; Rhymer & Simberloff, 1996; Todesco et al., 2016). Therefore, identification of the extent of hybridization and determination of the direction of introgression are critical to understand the evolutionary roles of hybridization in plants.

*Melastoma* L. (Melastomataceae), a shrub genus, comprises about 80–90 species in tropical Asia and Oceania (Wong, 2016). *Melastoma* has undergone rapid species radiation in the past 1 million years (Renner & Meyer, 2001). Hybridization is frequently reported amongst species pairs in this genus (Dai et al., 2012; Liu et al., 2014; Zhou et al., 2017; Zou et al., 2017). However, all these reported cases of hybridization in *Melastoma* were confined to species in China. As the species distribution center of *Melastoma*, Southeast Asia possesses about 70–80 species (Wong, 2016). So far, there have been no confirmed cases of hybridization in *Melastoma* in Southeast Asia, although several putative hybrids have been proposed based on morphology (Wong, 2015,2016). Borneo is the third biggest island in the world and the most in‐depth research in *Melastoma* has been conducted here (Meyer, 2001; Wong, 2016). According to Wong (2016), there are 41 species of *Melastoma* in Borneo, among which 40 species are endemic to this island. The remaining species, *M. malabathricum* L., is the most widespread species of *Melastoma*, occupying almost the whole range of this genus. The mechanism of high species diversity in *Melastoma* in Borneo (about 40% species of this genus occurring on this island) remains unclear. Natural hybridization may be an important mechanism for generating species diversity.

**Figure 1:**
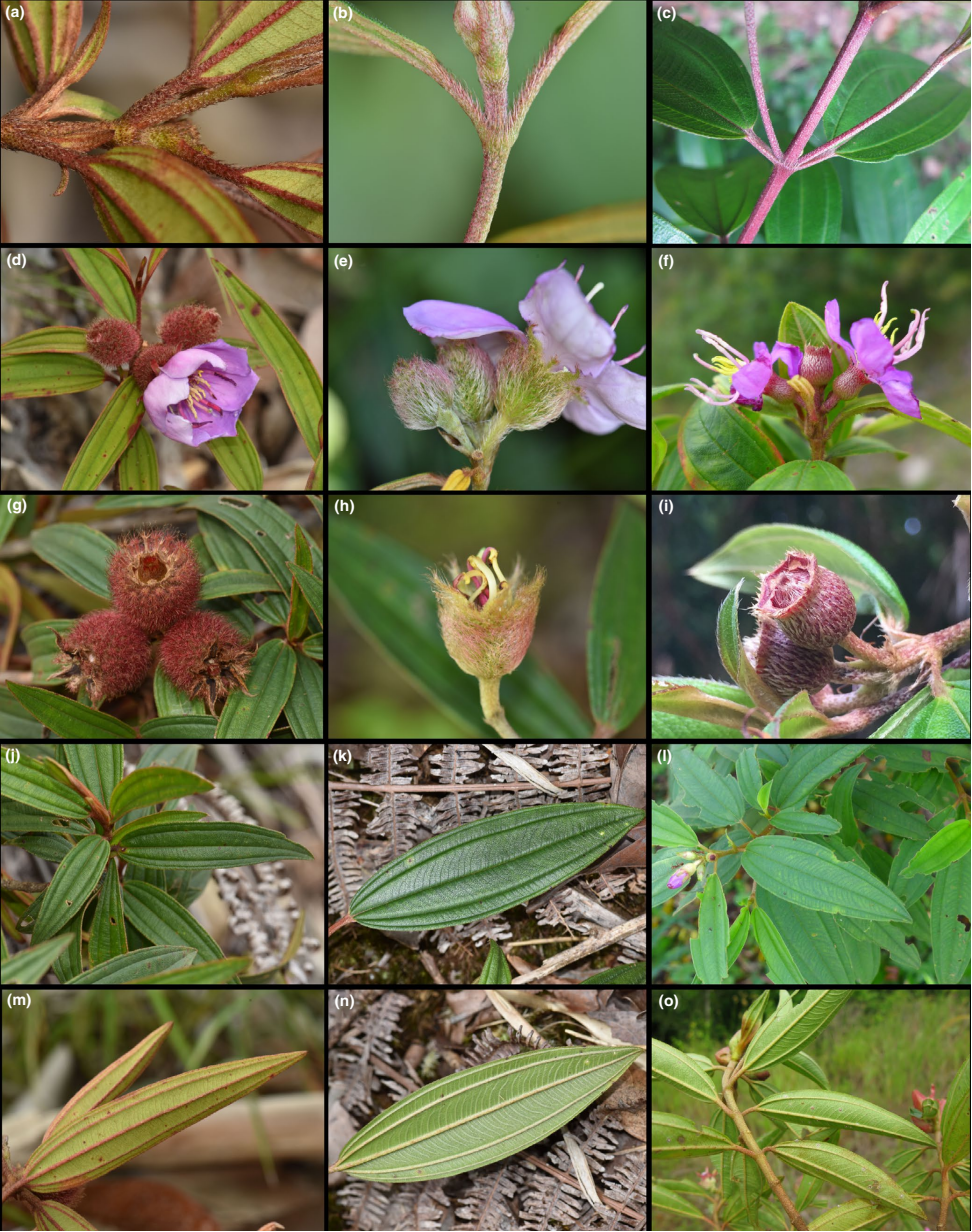
Morphological details of *Melastoma malabathricum* (the left column), the putative hybrid (the middle column), and *Melastoma beccarianum* (the right column) in Miri, Sarawak, Malaysia. Shown in the five rows from top to bottom are young twigs, hypanthium (with petals), hypanthium (after petal shedding), leaf (upper surface), and leaf (lower surface). *Melastoma beccarianum* and *M. malabathricum* are covered by penicillate emergences with long spreading bristles and short imbricate or appressed lanceolate scales on their hypanthiums, respectively, while the putative hybrid is covered by long branched appressed scales on its hypanthiums. Young twigs of *M. malabathricum* were covered with appressed scales, while those of both *M. beccarianum* and the putative hybrid were covered with slightly spreading scales. Moreover, *M. beccarianum* and *M. malabathricum* have lanceolate and ellipse leaves, respectively, while the putative hybrid has lanceolate‐ellipse leaves

During a field survey in Borneo, we found some putative hybrid individuals between *M. malabathricum* and *M. beccarianum* Cogn. along a roadside near Lambir Hills National Park, Miri, Sarawak, Malaysia. At this location, *M. malabathricum* and *M. beccarianum* coexist and the putative hybrid shows morphological intermediacy between the two species (Figure 1). Hypanthium indumentum is the most important trait to distinguish species of *Melastoma* (Meyer, 2001; Wong, 2016) and the two species indeed differ markedly in this aspect. *Melastoama beccarianum* and *M. malabathricum* are covered by penicillate emergences with long spreading bristles and short imbricate or appressed lanceolate scales on their hypanthiums, respectively, while the putative hybrid is covered by long branched appressed scales on its hypanthiums. Moreover, *M. beccarianum* and *M. malabathricum* have lanceolate and ellipse leaves, respectively, while the putative hybrid has lanceolate‐ellipse leaves. As hybrid identification based on morphology alone is not always reliable, we aimed to test the hypothesis of natural hybridization between *M. malabathricum* and *M. beccarianum* by molecular means. Previous studies showed that sequences of low‐copy nuclear genes were very useful for hybrid identification in *Melastoma* (Dai et al., 2012; Zhou et al., 2017; Zou et al., 2017), and we used this approach in this study.

## 2 MATERIALS AND METHODS

### 2.1 Plant materials

We sampled 19 individuals of *M. malabathricum* , 16 of *M. beccarianum,* and 16 of their putative hybrid in 2016 along a roadside near Lambir Hills National Park, Miri, Sarawak, Malaysia. In addition, 23 individuals of *M. malabathricum* were sampled from a natural population in University Malaysia Sabah, Sabah, Malaysia, and considered as a “pure” population because there is no other species occurring there. However, no “pure” populations of *M. beccarianum* were found, given that they are always sympatric with *M. malabathricum* or other congeneric species. We aimed to use this “pure” population to distinguish introgression from incomplete lineage sorting (ILS) of ancestral polymorphisms. For each individual, we collected one or two leaves and dried them with silica gel for subsequent DNA extraction.

### 2.2 DNA extraction, PCR, and sequencing

We used CTAB method (Doyle & Doyle, 1987) to extract DNA from dried leaves. Six nuclear genes adopted in previous studies (Chao et al., 2014; Dai et al., 2012; Huang et al., 2017; Zou et al., 2017) were tested in the three taxa and three nuclear genes (*gbss, vr,* and* tpi*) showed successful PCR amplification in all three taxa. *Gbss*, *tpi,* and *vr* encode granule‐bound starch synthase, triose phosphate isomerase, and vacuolar invertase, respectively. We conducted PCR amplification with the EasyTaq DNA polymerase (Transgen Biotech, Beijing, China) or the KOD‐FX DNA polymerase (TOYOBO, Osaka, Japan). PCR was conducted with the following conditions for all three nuclear genes: 94°C (4 min); 30 cycles of 94°C (40 s), 55°C (1 min), and 72°C (1 min); and a final extension of 8 min at 72°C The PCR products were purified by electrophoresis through a 1.2% agarose gel followed by use of the Pearl Gel Extraction Kit (Pearl Bio‐tech, Guangzhou, China). For sequences that contained more than one polymorphic site, cloning sequencing was performed using the pMD‐18T Vector Kit (Takara, Dalian, China). At least six positive clones were sequenced to phase the haplotypes of each sample. In addition, a chloroplast intergenic spacer (*trn*H‐*psb*A) was also amplified and sequenced using the same protocol as the nuclear genes. All sequences in this study have been deposited in GenBank with the accession numbers MH910371‐MH910491.

### 2.3 Sequence analyses

SeqMan (DNASTAR, Madison, WI, USA) was used to assemble and edit the sequences. These sequences were further aligned using Clustal X (Thompson, Gibson, Plewniak, Jeanmougin, & Higgins, 1997). DNASP 5.0 (Librado & Rozas, 2009) was applied to phase the haplotypes and calculate the nucleotide diversity as well as Tajima's *D* for each population. Haplotype network for each gene was constructed by Network v.4.6 (www.fluxus-engineering.com) with the median‐joining algorithm (Bandelt, Forster, & Röhl, 1999). Genomic admixture proportions of all individuals of the three taxa were assessed using the program Structure (Pritchard, Stephens, & Donnelly, 2000) with the default settings and employing the admixture model with correlated allele frequencies, as performed in Liao et al. (2015).

## 3 RESULTS

### 3.1 Sequence analysis of the partial *tpi* gene in *M. malabathricum* , *M. beccarianum,* and their putative hybrid

**Figure 2:**
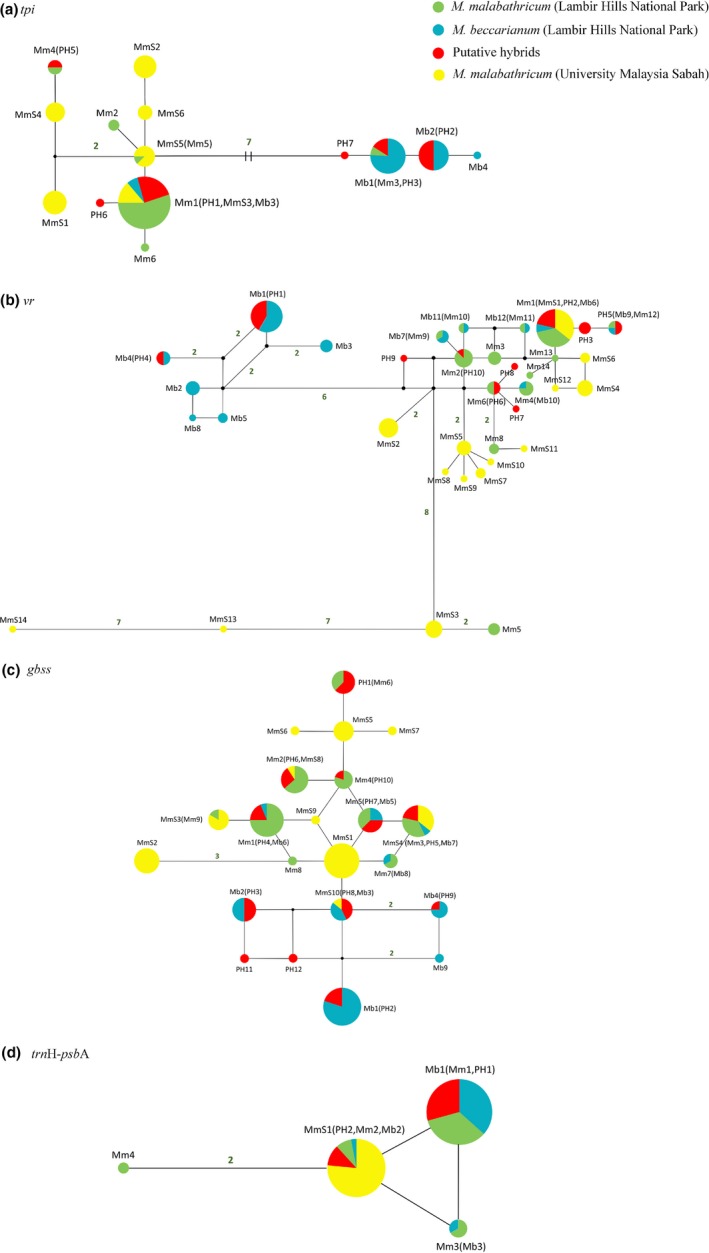
Median‐joining networks of (a) partial *tpi* gene, (b) partial *vr* gene, (c) partial *gbss* gene, and (d) chloroplast intergenic spacer *trn*H‐*psb*A. Haplotypes of each taxon are denoted using the first letter of its species name (“Mm” and “Mb” refer to *Melastoma malabathricum* and *Melastoma beccarianum* from Lambir Hills National Park, respectively; while “PH” and “MmS” represent the putative hybrid and allopatric *M. malabathricum* from University Malaysia Sabah) followed by a number ordered by quantity each population owned. Small black circles represent hypothetical haplotypes. Mutational steps are shown by the number near the connecting lines, and the number is omitted for those with only one mutational step

The partial *tpi* gene of *M. malabathricum*, *M. beccarianum,* and their putative hybrid was 699 bp in length, containing 18 nucleotide substitutions and two 1‐bp insertion/deletions. These variable sites generated 14 haplotypes (Figure 2a), among which four, six, six, and six haplotypes belong to* M. beccarianum*, the putative hybrid, *M. malabathricum* from Miri, and *M. malabathricum* from University Malaysia Sabah, respectively. Haplotype network of this gene could be divided into two clades, with seven mutational steps between them. One clade contained all haplotypes of the allopatric *M. malabathricum* population sampled from University Malaysia Sabah, most haplotypes of *M. malabathricum* from Miri, three haplotypes of the putative hybrid, and one low‐frequency haplotype of* M. beccarianum*; the other clade included two most common and one low‐frequency haplotypes of *M. beccarianum*, and three haplotypes of the putative hybrid. Four of six haplotypes of the putative hybrid were shared with either *M. beccarianum* or *M. malabathricum*, while two other low‐frequency haplotypes were private and only one mutational step away from the major haplotypes of its putative parents.

### 3.2 Sequence analysis of the partial *vr* gene in *M. malabathricum*, *M. beccarianum,* and their putative hybrid

After sequence alignment, the partial *vr* gene of the three taxa was 844 bp in length. This gene showed a high level of genetic variation, with 47 nucleotide substitutions that formed 36 haplotypes. For *M. malabathricum*, there were 14 and 13 haplotypes in the allopatric population from University Malaysia Sabah, and the population from Miri, respectively. Only the most common haplotype was shared between the two populations of *M. malabathricum*, suggesting pronounced differentiation at this gene between them. *Melastoma beccarianum* and the putative hybrid had 12 and 10 haplotypes, respectively. Six of the 10 haplotypes of the putative hybrid were shared with either *M. beccarianum* or *M. malabathricum*. The haplotype network of the *vr* gene fall into three haplotype groups, the first group contained six haplotypes of *M. beccarianum* and two haplotypes of the putative hybrid; the second group consisted of most haplotypes of *M. malabathricum*, eight haplotypes of the putative hybrid, and six low‐frequency haplotypes of* M. beccarianum*. The last group included four low‐frequency haplotypes of *M. malabathricum* (Figure 2b).

### 3.3 Sequence analysis of the partial *gbss* gene in *M. malabathricum*, *M. beccarianum,* and their putative hybrid

The aligned sequence of the partial *gbss* gene of the three taxa was 581 bp in length. A total of 20 nucleotide substitutions were detected and they generated 22 haplotypes. There were 10 haplotypes in the allopatric *M. malabathricum* population from University Malaysia Sabah. *Melastoma malabathricum*, *M. beccarianum,* and their putative hybrid sampled from Miri had nine, nine, and 12 haplotypes, respectively. Ten of 12 haplotypes of the putative hybrid were shared with those of either *M. beccarianum* or *M. malabathricum*, and only two low‐frequency haplotypes were private. Haplotype network of this gene could be divided into two clades. One clade contained all haplotypes of *M. malabathricum* (except MnS10) from University Malaysia Sabah and Miri, seven haplotypes of the putative hybrid (all shared with *M. malabathricum*) and five haplotypes of* M. beccarianum*; the other one was made up of six haplotypes, including three haplotypes shared by *M. beccarianum* and the putative hybrid, and two and one private to *M. beccarianum* and the putative hybrid, respectively (Figure 2c).

### 3.4 Sequence analysis of the chloroplast intergenic spacer *trn*H‐*psb*A in *M. malabathricum*, *M. beccarianum,* and their putative hybrid

The chloroplast intergenic spacer *trn*H‐*psb*A of the three taxa was 285 bp in length, containing two nucleotide substitutions and one 14‐bp triallelic insertion/deletion. The allopatric *M. malabathricum* from Sabah had only one haplotype, while *M. beccarianum, M. malabathricum*, and their putative hybrid from Miri had three, four, and two haplotypes, respectively (Figure 2d). As shown in the haplotype network, *M. beccarianum, M. malabathricum* shared three haplotypes, among which the two most common haplotypes were also shared with their putative hybrid.

### 3.5 Nucleotide diversity and structure analysis

At each of the three nuclear genes, the putative hybrid harbored higher nucleotide diversity (*π*) than either *M. malabathricum* or *M. beccarianum* (Table 1). Furthermore, the putative hybrid always had a positive value of Tajima's *D* at the three nuclear genes (significantly >0 at the *vr* gene).

**Table 1:** Nucleotide diversity (*π*) and Tajima's *D* at three nuclear genes of *M. beccarianum* (Mb), *M. malabathricum* (Mm), and their putative hybrid (PH)

		Nucleotide diversity (*π*)	Tajima's *D*
*tpi*	*Melastoma beccarianum* (Mb)	0.00379	−0.3098
	*Melastoma malabathricum* (Mm)	0.00245	−1.7540
	Putative hybrid (PH)	0.00876	1.8163
*vr*	*M. beccarianum* (Mb)	0.00811	0.7499
	*M. malabathricum* (Mm)	0.00562	−0.1329
	Putative hybrid (PH)	0.00976	2.5551***
*gbss*	*M. beccarianum* (Mb)	0.00452	−0.1213
	*M. malabathricum* (Mm)	0.00408	0.7053
	Putative hybrid (PH)	0.00659	0.3338

*** Represented the value of Tajima's *D* is significantly deviating from 0 (*p* < 0.01).

**Figure 3:**
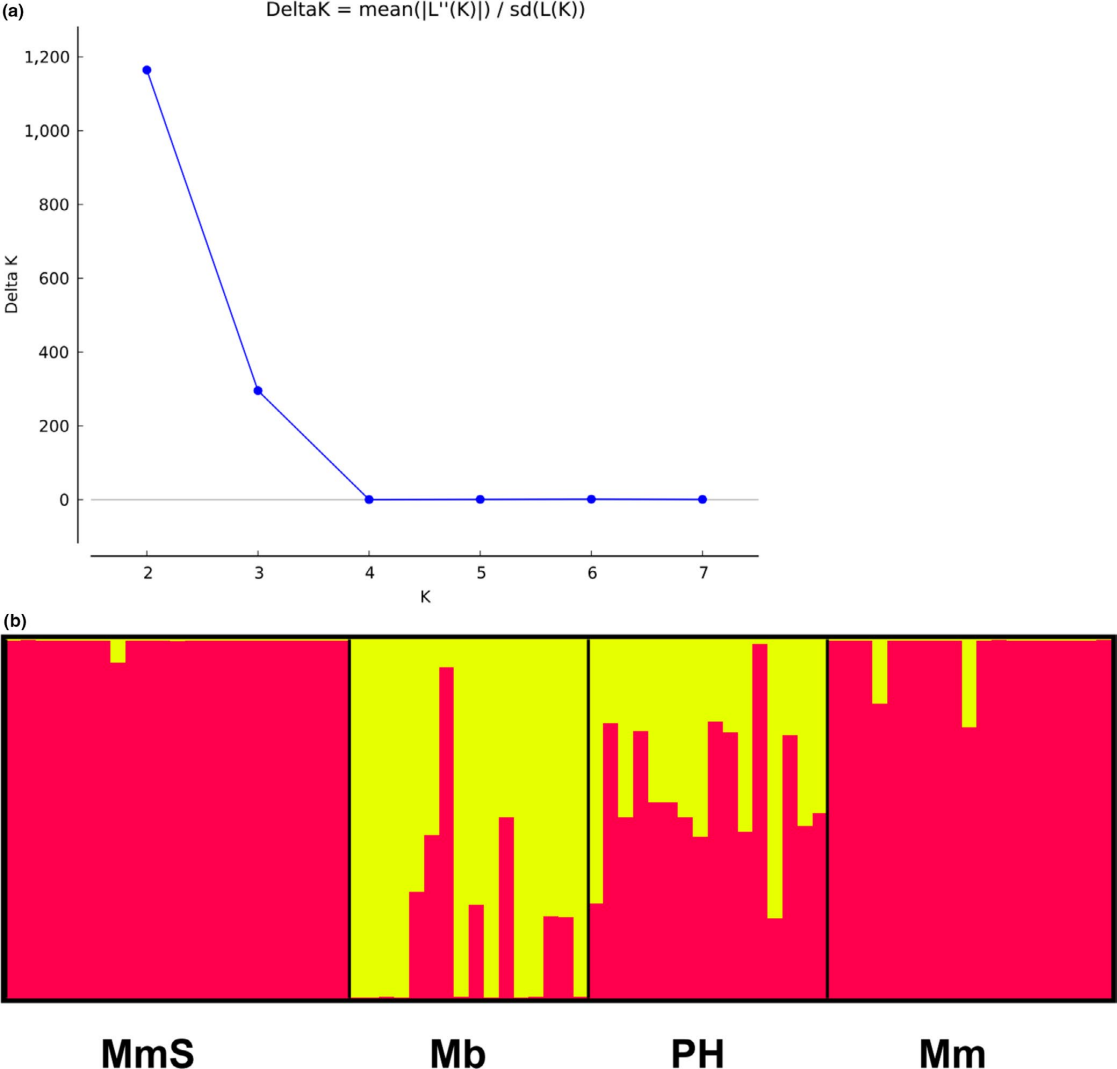
Clustering analysis results by STRUCTURE. (a) The best *K* value for three taxa of *Melastoma* by STRUCTURE based on sequence data of three nuclear genes. (b) Clustering analysis results with *K* = 3. Vertical bars represent individuals and probabilities of assignment to each cluster

The Structure analysis for the three taxa yielded a highest Δ*K* value for *K* = 2 (Figure 3a), indicating that two genetic clusters were most likely, corresponding to the two species. All the individuals of the putative hybrid showed an admixture of the two genetic clusters, supporting their hybrid status. The allopatric *M. malabathricum* population (MmS) appears “pure” except that one individual showed a low level of admixture (Figure 3b).

## 4 DISCUSSION

### 4.1 Molecular evidence for natural hybridization between *M. malabathricum* and *M. beccarianum*

In this study, we tested the hypothesis of natural hybridization between *M. beccarianum* and *M. malabathricum* by sequencing three nuclear genes and one chloroplast intergenic spacer. An allopatric population of *M. malabathricum* in University Malaysia Sabah was used to distinguish interspecific introgression from ILS of ancestral polymorphisms. At two of the three nuclear genes (*vr* and *tpi*), two major well separated haplogroups were detected, largely corresponding to each of the two putative parental species. At the third gene *gbss*, except for the haplotype MmS10, there are still two, albeit not well separated, haplogroups, corresponding to the two parental species. As the two clades have only one fixed nucleotide substitution, the haplotype MmS10 shared with *M. beccarianum* may result from homoplasy. There are two lines of evidence to prove that the suspected hybrid is indeed the hybrid between *M. beccarianum* and *M. malabathricum* from Lambir Hills National Park. First, morphological characteristics of the putative hybrid like leaf shape and hypanthium indumentum are intermediate between *M. beccarianum* and *M. malabathricum*. Second, analysis of haplotypes of all three nuclear loci shows that the putative hybrid has haplotypes of the two haplogroups corresponding to *M. beccarianum* and *M. malabathricum*, and most individuals of the putative hybrid have identical haplotypes with both *M. beccarianum* and *M. malabathricum* at one or more nuclear loci (Table 2). Moreover, higher nucleotide diversity and positive value of Tajima's *D* at all three nuclear genes in the putative hybrid suggested that it resulted from admixture of two divergent lineages. Because hybridization is an important mechanism for species diversification and pervasive in *Melastoma*, it is reasonable to suggest that hybridization should play an important role in species radiation in *Melastoma*.

**Table 2:** Genotype information of the putative hybrid (PH) between *M. beccarianum* (Mb) and *M. malabathricum* (Mm) at one chloroplast locus and three nuclear loci

Individual code	*trn*H‐*psb*A	*tpi*	*gbss*	*vr*
PH1	PH1 (Mm1, Mb1)	PH3 (Mb1, Mm3) PH7	PH5 (Mm3, Mb7, MmS10) PH9 (Mb4)	PH1 (Mb1) PH7
PH2	PH1 (Mm1, Mb1)	PH1 (Mm1, Mb3, MmS3) PH3 (Mm3, Mb1)	PH1 (Mm6) PH3 (Mb2)	PH2 (Mm1, Mb6, MmS1) PH2 (Mm1, Mb6, MmS1)
PH3	PH1 (Mm1, Mb1)	PH1 (Mm1, Mb3, MmS3) PH2 (Mb2)	PH1 (Mm6) PH8 (Mb3, MmS10)	PH3 PH6 (Mm6)
PH4	PH1 (Mm1, Mb1)	PH1 (Mm1, Mb3, MmS3) PH2 (Mb2)	PH5 (Mm3, Mb7, MmS10) PH11	PH1 (Mb1) PH3
PH5	PH1 (Mm1, Mb1)	PH1 (Mm1, Mb3, MmS3) PH5 (Mm4)	PH6 (Mm2, MmS8) PH10 (Mm4)	PH3 PH9
PH6	PH1 (Mm1, Mb1)	PH2 (Mb2) PH2 (Mb2)	PH2 (Mb1) PH12	PH1 (Mb1) PH2 (Mm1, Mb6, MmS1)
PH7	PH1 (Mm1, Mb1)	PH2 (Mb2) PH6	PH4 (Mm1, Mb6) PH7 (Mm5, Mb5)	PH1 (Mb1) PH10 (Mm2)
PH8	PH2 (Mm2, Mb2, MmS1)	PH1 (Mm1, Mb3, MmS3) PH2 (Mb2)	PH3 (Mb2) PH7 (Mm5, Mb5)	PH1 (Mb1) PH2 (Mm1, Mb6, MmS1)
PH9	PH1 (Mm1, Mb1)	PH3 (Mm3, Mb1) PH5 (Mm4)	PH2 (Mb1) PH7 (Mm5, Mb5)	PH1 (Mb1) PH2 (Mm1, Mb6, MmS1)
PH10	PH1 (Mm1, Mb1)	PH1 (Mm1, Mb3, MmS3) PH1 (Mm1, Mb3, MmS3)	PH4 (Mm1, Mb6) PH4 (Mm1, Mb6)	PH1 (Mb1) PH5 (Mm12, Mb9)
PH11	PH2 (Mm2, Mb2, MmS1)	PH1 (Mm1, Mb3, MmS3) PH2 (Mb2)	PH3 (Mb2) PH6 (Mm2, MmS8)	PH1 (Mb1) PH2 (Mm1, Mb6, MmS1)
PH12	PH1 (Mm1, Mb1)	PH1 (Mm1, Mb3, MmS3) PH2 (Mb2)	PH1 (Mm6) PH8 (Mb3, MmS10)	PH5 (Mm12, Mb9) PH6 (Mm6)
PH13	PH1 (Mm1, Mb1)	PH1 (Mm1, Mb3, MmS3) PH2 (Mb2)	PH1 (Mm6) PH2 (Mb1)	PH2 (Mm1, Mb6, MmS1) PH4 (Mb4)
PH13	PH1 (Mm1, Mb1)	PH1 (Mm1, Mb3, MmS3) PH2 (Mb2)	PH1 (Mm6) PH2 (Mb1)	PH2 (Mm1, Mb6, MmS1) PH4 (Mb4)
PH14	PH1 (Mm1, Mb1)	PH1 (Mm1, Mb3, MmS3) PH2 (Mb2)	PH1 (Mm6) PH2 (Mb1)	PH2 (Mm1, Mb6, MmS1) PH4 (Mb4)
PH15	PH2 (Mm2, Mb2, MmS1)	PH1 (Mm1, Mb3, MmS3) PH2 (Mb2)	PH3 (Mb2) PH6 (Mm2, MmS8)	PH1 (Mb1) PH2 (Mm1, Mb6, MmS1)
PH16	PH2 (Mm2, Mb2, MmS1)	PH1 (Mm1, Mb3, MmS3) PH3 (Mm3, Mb1)	PH5 (Mm3, Mb7, MmS10) PH8 (Mb3, MmS10)	PH1 (Mb1) PH8

Note

PH1–PH16 in the first column are 16 individuals of the putative hybrid (PH). The code of each haplotype is the same as that in Figure 2. Haplotypes in brackets are the haplotypes of *M. beccarianum* (Mb), *M. malabathricum* from Miri (Mm), and *M. malabathricum* from University Malaysia Sabah (MmS).

*M. beccarianum*: *Melastoma beccarianum*; *M. malabathricum*: *Melastoma malabathricum*.

### 4.2 Bidirectional introgression between *M. malabathricum* and *M. beccarianum*

We observed haplotype sharing between *M. beccarianum* and *M. malabathricum* at all three nuclear genes and one chloroplast intergenic spacer. Interspecific haplotype sharing can be caused by introgression and ILS of ancestral polymorphisms. In this study, we have an allopatric population of *M. malabathricum* that can be used to distinguish them. If ILS holds here, we would expect a comparable extent of haplotype sharing between *M. beccarianum* and either symaptric *M. malabathricum* from Miri or allopatric *M. malabathricum* from Sabah. However, at the* tpi* and* vr* genes, the frequency of haplotype sharing between *M. beccarianum* and symaptric *M. malabathricum* is much higher than that between *M. beccarianum* and allopatric *M. malabathricum* (22:4 for *tpi* and 9:2 for *vr*; Table 3). At the *gbss* gene, the frequency of haplotype sharing in the former is slightly higher than that in the latter (5:4). This suggests that haplotype sharing between symaptric *M. beccarianum* and *M. malabathricum* should stem from introgression rather than ILS. Our data also show that bidirectional introgression happens between *M. beccarianum* and *M. malabathricum* (*tpi*) and introgression is asymmetrical, mainly from *M. malabathricum* to *M. beccarianum* (*vr* and *gbss*).

**Table 3:** Genotype information of *M. beccarianum* (Mb) at four loci. Haplotypes in the brackets means the haplotypes of *M. beccarianum* (Mb) shared with the haplotypes in Miri *M. malabathricum* (Mm) population or Sabah *M. malabathricum* (MmS) population or both

Individual code	*trn*H‐*psb*A	*tpi*	*gbss*	*vr*
Mb1	Mb1 (Mm1)	Mb1 (Mm3) Mb2	Mb1 Mb9	Mb1 Mb1
Mb2	Mb1 (Mm1)	Mb1 (Mm3) Mb1 (Mm3)	Mb2 Mb3 (MmS10)	Mb1 Mb1
Mb3	Mb1 (Mm1)	Mb1 (Mm3) Mb3 (Mm1, MmS1)	Mb6 (Mm1) Mb4	Mb6 (Mm1, MmS1) Mb3
Mb4	Mb1 (Mm1)	Mb1 (Mm3) Mb4	Mb1 Mb2	Mb1 Mb1
Mb6	Mb1 (Mm1)	Mb2 Mb2	Mb1 Mb3 (MmS10)	Mb1 Mb8
Mb7	Mb1 (Mm1)	Mb1 (Mm3) Mb1 (Mm3)	Mb1 Mb1	Mb9 (Mm12) Mb1
Mb8	Mb1 (Mm1)	Mb1 (Mm3) Mb2	Mb1 Mb1	Mb1 Mb6 (Mm1, MmS1)
Mb9	Mb1 (Mm1)	Mb1 (Mm3) Mb2	Mb1 Mb2	Mb2 Mb3
Mb10	Mb1 (Mm1)	Mb1 (Mm3) Mb1 (Mm3)	Mb1 Mb1	Mb1 Mb3
Mb11	Mb1 (Mm1)	Mb1 (Mm3) Mb1 (Mm3)	Mb1 Mb3 (MmS10)	Mb2 Mb2
Mb12	Mb1 (Mm1)	Mb2 Mb2	Mb1 Mb2	Mb1 Mb1
Mb13	Mb1 (Mm1)	Mb2 Mb2	Mb1 Mb7 (Mm3, MmS4)	Mb2 Mb12 (Mm11)
Mb14	Mb1 (Mm1)	Mb1 (Mm3) Mb3 (Mm1, MmS1)	Mb5 (Mm5) Mb4	Mb1 Mb7 (Mm9)
Mb15	Mb1 (Mm1)	Mb3 (Mm1, MmS1) Mb3 (Mm1, MmS1)	Mb1 Mb5 (Mm5)	Mb7 (Mm9) Mb11 (Mm10)
Mb16	Mb1 (Mm1)	Mb1 (Mm3) Mb1 (Mm3)	Mb1 Mb4	Mb7 (Mm9) Mb11 (Mm10)
Mb17	Mb2 (Mm2, MmS1)	Mb1 (Mm3) Mb1 (Mm3)	Mb1 Mb8 (Mm7)	Mb1 Mb4

*M. beccarianum*:* Melastoma beccarianum*;* M. malabathricum*:* Melastoma malabathricum*.

### 4.3 Factors contributing to natural hybridization between *M. beccarianum* and *M. malabathricum*

Several factors may contribute to natural hybridization between *M. beccarianum* and *M. malabathricum*. First, species of *Melastoma* diverged in a relatively short evolutionary time and reproductive isolation between them is still incomplete. It is reported that *Melastoma* has undergone a rapid species radiation, with about 80–90 species formed in the past 1 million years (Renner & Meyer, 2001). This is also supported by our sequence data, for example, *M. beccarianum* and *M. malabathricum* can not be distinguished by the chloroplast intergenic spacer and they are very close at the* gbss* gene. Second, overlapping geographical distribution and flowering time, and shared pollinators provide the chance to hybridize. *Melastoma beccarianum* is endemic to northern Borneo, while *M. malabathricum* is the most widespread species of *Melastoma*. Overlapping geographical distribution between them is expected in some places of northern Borneo, like the one found in this study. Both species flower throughout the year, and pollinators like bumble bees are largely shared among species of *Melastoma* (Gross, 1993; Loh, 2008; Luo, Zhang, & Renner, 2008), offering ample opportunities for hybridization.

### 4.4 Conservation implications for rare species in *Melastoma*

While *M. malabathricum* is the most widespread species of *Melastoma*,* M. beccarianum* is endemic to northern Borneo, and found across west Sabah, Brunei, and north Sarawak (Wong, 2016). *Melastoma malabathricum* could occurs in all types of dryland areas throughout Borneo including on ultramafic rocks and up to mid‐montane elevation, while *M. beccarianum* mainly occurs in lowland areas, especially on substrates derived from sedimentary rocks, which is a relatively limited habitat type, resulting in scattered distribution and relatively small population size. Consistent with this, *M. malabathricum* harbors much higher nucleotide and haplotype diversity than* M. beccarianum* at all three nuclear genes. As genetic variation is necessary for species survival and adaptation to changing environments, lower genetic variation of a species may pose more risk of extinction.

Hybridization is a double‐edge sword: it may drive rare taxa to extinction through genetic and demographic swamping (Huxel, 1999; Rhymer & Simberloff, 1996; Todesco et al., 2016); conversely, a net fitness can be gained to one or both taxa without loss of species integrity by adaptive trait transfer between species by introgression (Abbott et al., 2016; Lamichhaney et al., 2016; Zhang et al., 2016).

Based on our results, bidirectional but asymmetrical introgression occurs between *M. beccarianum* and *M. malabathricum*, mainly from the common species *M. malabathricum* to the relatively rare species *M. beccarianum*. This implies repeated backcrossing to *M. beccarianum* following hybridization with *M. malabathricum*. A previous study showed that, when nuclear introgression is from the common species to the rare species, there is more risk of extinction than the opposite direction (Todesco et al., 2016). Thus, introgression to *M. beccarianum* from the common species *M. malabathricum* may incur the risk of extinction of *M. beccarianum*. On the other hand, hybridization can lead to a waste of reproductive energy of *M. beccarianum*, which can lead to a decline in its population size, further reducing the genetic variation and thus increasing the risk of extinction. However, there is also potential adaptive gene transfer from *M. malabathricum* to *M. beccarianum*, which may benefit population growth of *M. beccarianum* because *M. malabathricum* can adapt to a variety of habitats. Long‐term monitoring on the hybrid zones of *M. beccarianum* and *M. malabathricum* is needed to assess the fate of *M. beccarianum* in the context of hybridization.

*Melastoma beccarianum* and *M. malabathricum* have ecological isolation in terms of soil substrates and light intensity: *M. malabathricum* prefers acid soil and open habitats, while *M. beccarianum* is usually found in forest edge with slight shade and substrates derived from sedimentary rocks. Habitat disturbance may increase the opportunity for hybridization and introgression, as seen in this study, hybrids are always found along the roadside where ecological barriers have been broken because of road construction. Therefore, to reduce or even avoid habitat disturbance should be the key to conserving *M. beccarianum* as well as other rare species of *Melastoma*.

## CONFLICT OF INTEREST

None declared.

## AUTHOR CONTRIBUTIONS

RW, PZ, and RZ designed the study. GT, ZH, YW, ZN, SH, and YL collected materials, which was then performed experiments by RW and PZ. PZ, GT, ZH, YW, and ZN guided the experiments. RW and PZ analyzed and interpreted the data and wrote the manuscript with guidance of RZ, WW, YL, and SH. All authors read and approved the final manuscript.

## Data Availability

The haplotype sequences of our study involved are deposited in GenBank with accession numbers MH910371–MH910491.
